# Assessing Self‐medication Practices Among Healthcare Providers With Migraine in Saudi Arabia

**DOI:** 10.1002/brb3.70724

**Published:** 2025-08-22

**Authors:** Bandar Nasser Aljafen, Jodi Mohamad Alkahwaji, Sarah Amin Alamoudi, Shaimaa Tawfik Jamous, Sara Mohammed Almesfer, Aljohrah Sultan Alanazi, Fatima Yahya Al‐Aidaros

**Affiliations:** ^1^ Neurology Unit, Department of Medicine, College of Medicine King Saud University Riyadh Saudi Arabia; ^2^ College of Medicine Dar Al Uloom University Riyadh Saudi Arabia

**Keywords:** self‐medication, migraine, healthcare providers, headache, medication‐overuse headache

## Abstract

**Objectives:**

Migraine is a neurological disorder characterized by recurrent headaches resulting from abnormal regulation of sensory input to the head. Self‐medication (SM) is a prevalent practice among healthcare providers (HCPs), with potentially detrimental consequences. This study aims to investigate SM practices among HCPs in Saudi Arabia, specifically focusing on their management of migraines.

**Methods:**

This cross‐sectional study was conducted in Riyadh, Saudi Arabia, using an electronic self‐administered questionnaire. This study encompassed various categories of healthcare professionals in Riyadh.

**Results:**

Among the 1054 participants, 165 healthcare professionals reported SM for migraine headaches. The primary reasons for this practice included perception of having sufficient information about the disease and its management (60.0%), ease of medication access (53.9%), and busy schedule (46.7%). The most commonly overused self‐prescribed medications for migraines were paracetamol (70.9%) and nonsteroidal anti‐inflammatory drugs (NSAIDs) (43.0%), both classified as over the counter medication. Out of the 165 self‐medicating HCPs with migraines, approximately half (53.2%) reported that the medication was effective in the treatment of migraine, and the majority (78.5%) reported no side effects from self‐medication. The 55.8% of participants were unaware of the term “medication‐overuse headaches” (MOH). SM was significantly correlated with the specialty of the HCP (*p* < 0.001), as was perceiving headache as a migraine (*p* < 0.001). Significant positive correlations were observed between certain SM drugs and the reported side effects.

**Conclusions:**

A high prevalence of headache was observed among HCPs in Saudi Arabia. Perceived sufficient information about the disease and its management, accessible medications, and a busy schedule were among the most common reasons for SM among HCPs with migraines. Moreover, most participants were unfamiliar with the term MOH and needs immediate attention and implement targeted policies and educational programs, including mandatory continuing medical education (CME) focused on MOH diagnosis, and management. Additional studies are required to identify other contributing factors or unintended complications associated with SM among healthcare professionals.

## Graphical abstract

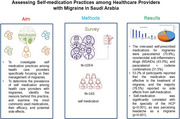

## 1 Introduction

Migraine is a neurological disorder involving altered regulation and control of afferents, with a particular focus on the cranium. The key marker of migraine is repeated episodes of headache. The estimated global prevalence of migraine is 14%–15%, and migraine accounts for 4.9% of global ill health (Steiner and Stovner 2023). The overall prevalence of migraine in the US population ranges from 11.7% to 14.7%, which has remained consistent for the past 30 years: 17.1% to 19.2% in females and 5.6% to 7.2% in males (Cohen et al. 2024). The prevalence of migraine headaches among the Saudi population is 37.2%, with a higher prevalence among females (81.1%) (Bamalan et al. 2021). A recent study among physicians in Saudi Arabia reported the prevalence of migraines was 17.1%, with 6.823 as the average number of headache attacks per month (Algahtani et al. 2024).

Migraine disorders are associated with a wide range of risk factors, including demographic factors (age, sex), biological factors (hormonal imbalances, metabolic, and genetic factors), psychological factors (stress, depression, anxiety disorders, etc.), and external or environmental factors (climate change, smoking, and alcohol consumption, etc.) (Amiri et al. 2022). Headaches were identified as some of the most common symptoms reported by office workers. The frequent incidence of headache among office workers significantly impairs their quality of life and productivity, leading to economic burdens. The most common factors associated with headache in office workers include exposure to light, heat, drafts, odors, poor air quality in the work environment, and day and night shift duties related to sleep disorders (Appel et al. 2020). Mental and social factors are also associated with work‐related headaches, including stress due to workload, lack of autonomy, and job dissatisfaction, etc. (Magnavita et al. 2021; Magnavita et al. 2020).

Healthcare providers (HCPs) experience high incidences of migraine. This is largely attributed to their extended working hours and regular night shifts (Al Maqwashi et al. 2024; Alturaiki et al. 2023). Furthermore, HCPs have been found to be at an increased risk of frequent migraine attacks compared to the general population (Kuo et al. 2015). Headaches among healthcare professionals should not be neglected as they can act as early indicators of serious conditions, such as the onset of ischemic stroke or cerebral venous sinus thrombosis (Alshurafa et al. 2018; Lebedeva et al. 2020)

Self‐medication (SM), or the self‐administration of medicine, is prevalent globally, including in Saudi Arabia (Al‐Ghamdi et al. 2020). Individuals, families, and communities often use medications to treat health conditions or symptoms without a prescription or diagnosis from trained HCPs (Onchonga et al. 2020). Common factors contributing to SM in HCPs include knowledge and availability of prescribed medications, excessive workload, and concerns about privacy and confidentiality (Babatunde et al. 2016; Rosen et al. 2000). While healthcare providers’ experience allows them to utilize medications differently from the general public, SM poses significant risks, including addiction, decreased functionality, unprofessional conduct, and potential ties to illegal activities. Additionally, SM among HCPs can lead to medical malpractice, carelessness, and lack of objectivity in diagnosis and treatment (Fekadu et al. 2020). Given the direct impact of HCP's well‐being on patient care quality and their own health, this study aimed to investigate SM practices among HCPs in Saudi Arabia, specifically focusing on their management of migraines. In addition, we aimed to determine the prevalence of SM among HCPs with migraines, identify the reasons behind this practice, and examine the most commonly used medications, their efficacy, and potential side effects.

## 2 Materials and Methods

### 2.1 Ethical Consideration

This cross‐sectional study was conducted in Riyadh, Saudi Arabia, between February 1, 2022, and December 31, 2022, and was approved by the Institutional Review Board (IRB) of the College of Medicine, King Saud University, Kingdom of Saudi Arabia (IRB Approval No. E‐22‐6683). The study was conducted in accordance with the ethical standards of the Declaration of Helsinki.

### 2.2 Study Design and Participants

This study was used an electronic self‐administered questionnaire adopted with permission and modification from a prior research project conducted in Italy (Brusa et al. 2019). The study inclusion criteria include, participants were HCPs, including physicians, dentists, nurses, pharmacists, and paramedics at primary, secondary, and tertiary hospitals in Riyadh, Saudi Arabia. The inclusion criteria were as follows: being a licensed healthcare professional, a migraineur within the age range of 20–65 years, self‐medicating, and currently practicing in Riyadh, Saudi Arabia. The survey was distributed across various healthcare facilities including primary, secondary, and tertiary hospitals at Riyadh, Saudi Arabia. Distribution methods included manual dissemination via scannable QR codes. Prior to dissemination, all distribution efforts were conducted with the explicit permission of relevant institutional authorities. The high‐quality printed materials that displayed unique Quick Response (QR) codes, or unique QR codes displayed on mobiles, linked to Google Doc forms and were manually distributed directly to targeted HCPs at healthcare facilities (primary, secondary, and tertiary hospitals).

Modifications to the questionnaire included the addition of sections addressing healthcare professional (HCP) specialty, type of institute, family history of migraine, taking SM for migraine, frequency of intake of self‐medication, effectiveness of the medications, taking self‐prophylaxis for migraine, familiar with the term “medication‐overuse headache”, experienced side effects from self‐medication, etc. The modified survey instrument underwent a rigorous, multi‐stage validation and refinement process to ensure its clarity, relevance, and accuracy. Initially, the modified survey instrument was subjected to a comprehensive review by a panel of subject matter experts. Following the expert review and subsequent refinement, the survey instrument was pilot‐tested with a small group (*n* = 30) drawn from the target population, aimed to identify any unclear questions or irrelevant items, that may have been missed in earlier stages. The modified questionnaire was structured into eight distinct sections: (1) Consent and Demographic Data: Collection of participant consent and basic demographic information. (2) ID‐Migraine Screener Test: A validated tool utilized for the identification of migraineurs and the exclusion of non‐migraineur cephalalgic patients. (3) SM Query: A specific question to include only healthcare professionals (HCPs) who self‐medicated for their headaches, thereby measuring the prevalence of this practice. (4) Reasons for Self‐Medication: Questions inquiring about the underlying motivations for self‐medication. (5) Medication Types: Assessment of the specific types of medications used for self‐treatment. (6) Treatment Efficacy and Prophylaxis: Evaluation of the perceived efficacy of acute and prophylactic therapies. (7) Side Effects: Documentation of any adverse effects associated with the aforementioned medications. (8) Knowledge Assessment: A section designed to assess participants' familiarity with and comprehension of the term “medication‐overuse headache.”

Migraine diagnosis within this study was established using the ID‐Migraine Screener test. This screener comprises three questions:

Did you have the following with your headaches?

➢ You felt nauseated or sick to your stomach? Yes/No
➢ Light bothered you (a lot more than when you did not have headaches)? Yes/No
➢ Your headaches limited your ability to work, study, or do what you needed to do for at least 1 day? Yes/No

For the purpose of this study, participants who provided a “yes” response to two or three of these ID‐Migraine Screener questions were operationally defined and identified as “migraine sufferers.” This approach aligns with the ID‐Migraine Screener's demonstrated high sensitivity and specificity as a screening tool, typically indicating a 75%–93% probability of migraine when compared to a clinical diagnosis using the International Classification of Headache Disorders (ICHD) criteria (Olesen 2018).

The sample size calculation was done using the formula for estimating a population proportion: *n* = *Z* ^2^×*P*(1−*P*)/*d* ^2^, Z‐statistic (*Z*) at 1.96 (for a 95% confidence level), the expected prevalence (*P*) at 10% to maximize the sample size, and the precision (*d*) at 5%. The initial sample size was estimated to be 139 and was adjusted to 153 to compensate for the nonresponse rate.

Of the initially invited HCPs, 1171 consented to participate, with 1054 actively engaging with the survey. Among the 1054 participants, 803 had complained of headache during the past 3 months, and 219 individuals were identified as migraine sufferers based on the criteria of the ID‐Migraine Screening test. Among the individuals suffering from migraines, 165 HCPs who reported SM for their migraine headaches were selected for this study. The criteria used to establish the diagnosis of migraine and the demographic characteristics of the participants (*n* = 1054) are shown in Supporting Data 1 and Table S1. The selection of the study participants is shown in Supporting Data 2 and Figure S1.

### 2.3 Statistical Analysis

Descriptive statistics (percentages, means, and standard deviations) were used to identify the demographics and other characteristics of the study participants. Correlations among study outcomes were tested; Pearson's correlation was used to determine the correlation between continuous variables, while Spearman's correlation was used to determine the correlation when one variable was ordinal. Data are mean ± standard deviation, unless otherwise stated. Significant positive correlations with self‐medications and the reported side effects was analyzed using Chi‐square test. The results were considered significant at *p* < 0.05. Data were analyzed using the statistical program SPSS (IBM SPSS Statistics for Windows, V23.0, Armonk, NY, USA version 23.0).

## 3 Results

### 3.1 Demographic Characteristics of Study Participants

Among the participants, those who actively engaged in the survey (*N* = 1054) had a mean age of 34.04 ± 8.2 years; 46.5% were male and 53.5% were female. More than 50% of participants were specialty nurses (32.5%) or physicians (24.6%). Among 1054 participants, 803 (76.2%) reported that they experienced headaches during the past 3 months, and 219 (20.8%) reported that they experienced more than two symptoms, including nausea, photophobia, or disabling headache (Supporting Data 1; Table S1).

The clinical and demographic characteristics of the 165 participants who reported headaches during the past 3 months, having more than two symptoms, including nausea, photophobia, or disabling headache, and self‐medicating for their migraine headaches, are shown in Table 1. The mean age of study participants was 33.41 ± 7.19 years; 28.5% were male and 71.5% were female. 41.2% of study participants were nurse specialists, and 28.4% were physicians. 48.5% of participants had a family history of migraine, and 36.4% had migraine for the previous 5 years. Among participants with migraine in the past 3 months, 66.7% experienced 1–5 migraine days (Table 1).

**TABLE 1:** Clinical and demographic characteristics of study participants reported self‐medicating for their migraine headaches.

Characteristic	Participants (*N* = 165)
Age, years (mean ± SD)	33.41 ± 7.19
Sex *N*(%)	
Male	47 (28.5)
Female	118 (71.5)
Specialty *N*(%)	
Physician	47 (28.4)
Dentist	9 (5.5)
Nurse	68 (41.2)
Pharmacist	9 (5.5)
Paramedic	32 (19.4)
Type of institute *N*(%)	
Primary healthcare center	46 (27.9)
Secondary healthcare center	30 (18.2)
Tertiary healthcare center	89 (53.9)
Have a family history of migraine? *N*(%)	
Yes	80 (48.5)
No	85 (51.5)
Duration of migraine *N*(%)	
≤1 year	50 (30.3)
4–5 years	60 (36.4)
6–9 years	29 (17.6)
≥10 years	26 (15.8)
Days of migraine episodes in the past 3 months *N*(%)	
1–5 days	110 (66.7)
6–10 days	28 (17)
11–15 days	15 (9.1)
16–20 days	3 (1.8)
21–25 days	4 (2.4)
26–30 days	2 (1.2)
≥31	3 (1.8)

### 3.2 Perceptions and Habits of SM of Migraine

**FIGURE 1:**
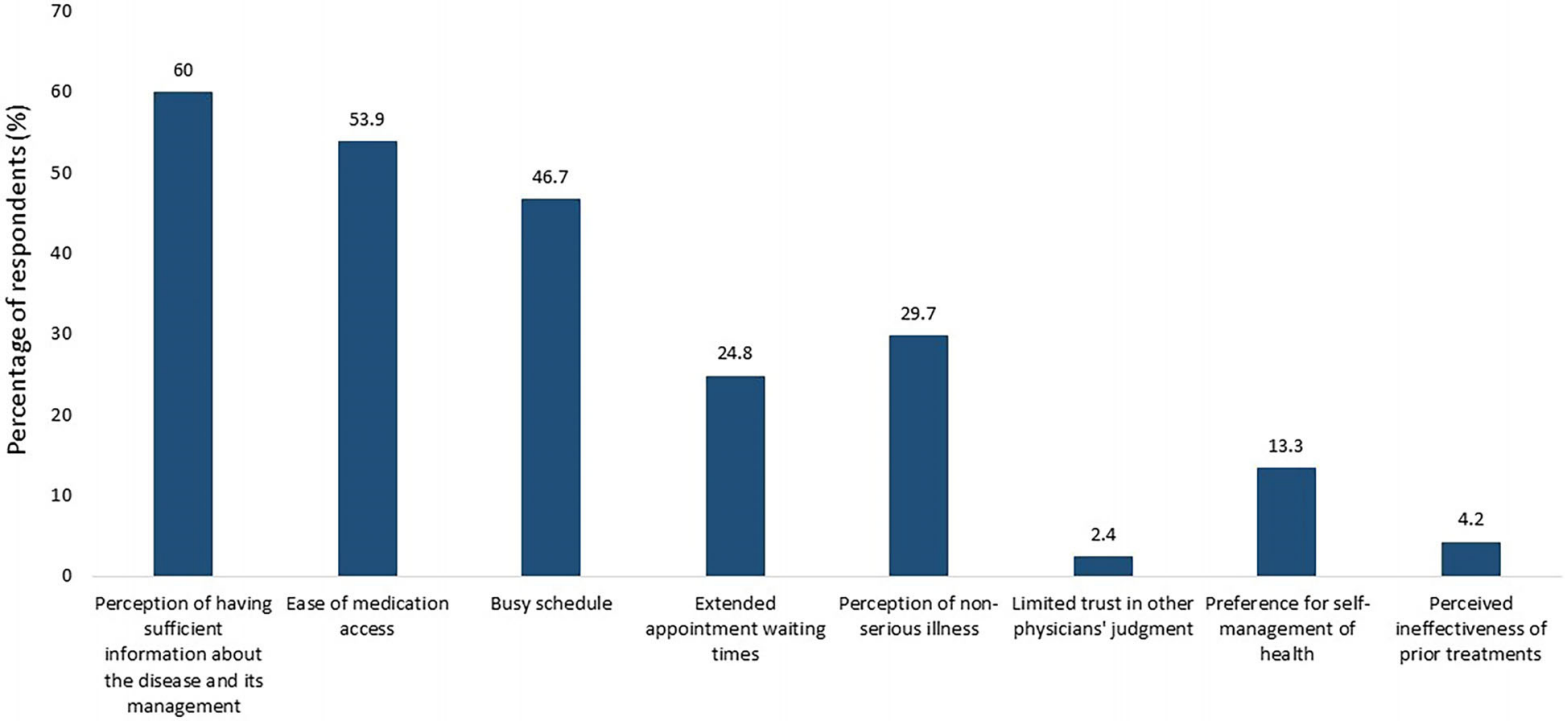
Reasons for taking self‐medication for treatment of migraine among participants (*N* = 165).

The perceptions and habits of SM of migraine among participants are shown in Table 2. The 60.6% of participants considered migraine an illness. The 70.3% of study participants took SM 1–3 days per month, and 50.9% reported that SM is effective for them. 55.8% of participants were unaware of the term “medication‐overuse headaches.” The reasons for SM as reported by the study participants are shown in Figure 1. The 60% of study participants had perception of having sufficient information about the disease and its management, 53.9% cited easy access to medication, and 46.7% attributed this to busy schedules.

**TABLE 2:** Perception and habits of self‐medication of migraine among participants.

	Participants (*N* = 165)
Regard headache as migraine? *N*(%)	
Yes	111 (67.3)
No	54 (32.7)
Consider migraine an illness? *N*(%)	
Yes	100 (60.6)
No	65 (39.4)
Treated by a medical professional before? *N*(%)	
Yes	41 (24.8)
No	124 (75.2)
Taking self‐medication for migraine? *N*(%)	
Yes	165 (100)
No	0 (0.0)
Frequency of intake of self‐medication? *N*(%)	
1–3 days/month	116 (70.3)
4–10 days/month	37 (22.4)
≥ 11 days/month	6 (3.6)
Missing	6 (3.6)
Effectiveness of the medications? *N*(%)	
Effective	84 (50.9)
Often effective	56 (33.9)
Rarely effective	13 (7.9)
Not effective	5 (3.0)
Missing	7 (4.2)
Taking self‐prophylaxis for migraine? *N*(%)	
Yes	21 (12.7)
No	136 (82.4)
Missing	8 (4.8)
Familiar with the term “medication‐overuse headache”? *N*(%)	
Yes	65 (39.4)
No	92 (55.8)
Missing	8 (4.8)

### 3.3 Correlation Between Different Factors and Migraine

Among the participants, only 21% reported the correct definition of the term “medication‐overuse headache” as headache occurring on 15 or more days per month in a patient with pre‐existing primary headache (Supporting Data 2; Figure S2). Regarding the demographics of the perception of headache and migraine, older participants had a greater tendency for perceiving headache as a migraine (*r_s_* = 0.163; *p* = 0.037) and were more likely to seek medical professional treatment (*r_s_* = 0.158; *p* = 0.042). Participants who had experienced a migraine headache for a longer duration had a greater tendency for perceiving headache as a migraine (*r_s_* = −0.402; *p* < 0.001) and perceiving migraine as an illness (*r_s_* = 0.254; *p* = 0.001). Moreover, they were more likely to seek medical professional treatment (*r_s_* = 0.267; *p* = 0.001). Presence of a family history of migraine was also significantly positively correlated with perceiving headache as a migraine (*r_s_* = 0.186; *p* = 0.014). Participants who took SM more frequently also tended to perceive headache as a migraine (*r_s_* = 0.169; *p* = 0.033) and migraine as an illness (*r_s_ =* 0.185*; p* = 0.019). They also tended to experience longer durations (*r_s_* = 0.193; *p* = 0.015), with more frequent monthly attacks (*r_s_* = 0.500; *p* < 0.001). Interestingly, participants who perceived headache as a migraine (*r_s_* = −0.274; *p* = 0.001), migraine as an illness (*r_s_* = ‐0.300; *p* < 0.001), and had more frequent monthly attacks of migraine (*r_s_* = −0.222; *p* = 0.005) were inversely correlated with the effectiveness of SM. Regarding prophylaxis of migraine, there was a significant positive correlation with the duration of migraine (*r_s_* = 0.200; *p* = 0.013), frequency of attacks (*r_s_* = 0.162; *p* = 0.043), and reporting of medical professional consultation for treatment (*r_s_* = 0.405; *p* < 0.001) (Supporting Data 1; Table S2).

### 3.4 Medications and Side Effects

The relative frequencies of the side effects of the medications used by the study participants are shown in Table 3. 75.2% of study participants experience no side effects from SM for migraines. The most commonly reported side effect of migraine SM was nausea, followed by stomachache.

**TABLE 3:** Side effects self‐medication of migraine.

Experienced side effects from self‐medication? *N*(%)	
Yes	34 (20.6)
No	124 (75.2)
Missing	7 (4.2)
Reported side effects (*N* = 34)	
Nausea	21
Stomach ache	12
Vomiting	7
Dizziness	7
Drowsiness	7
Heartburn	6
Palpitations	6
Constipation	3
Diarrhea	3
Numbness	3
Allergies	2
Itchiness	1
Others	2

**FIGURE 2:**
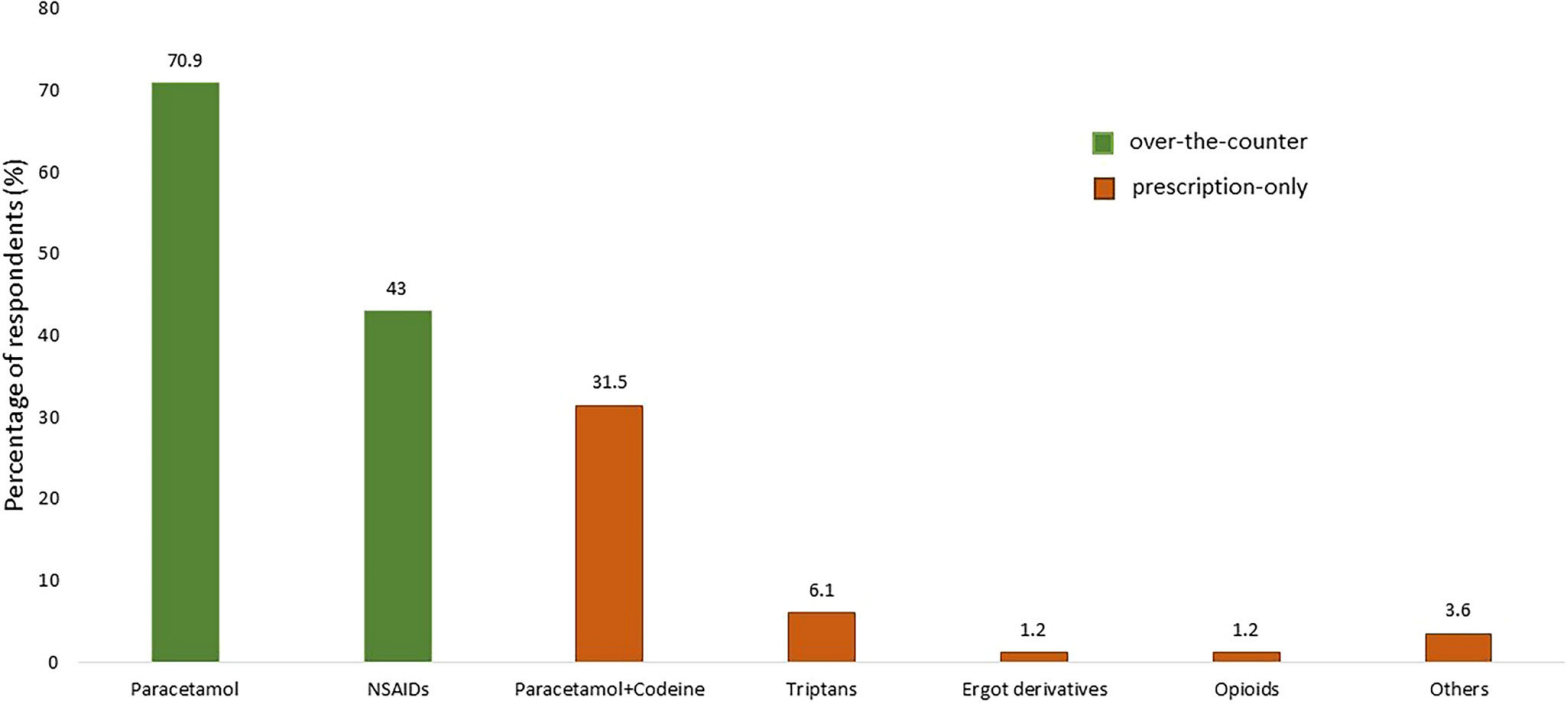
Medications (over‐the‐counter and prescriptions‐only) used for self‐medication in migraine among participants (*N* = 165).

**FIGURE 3:**
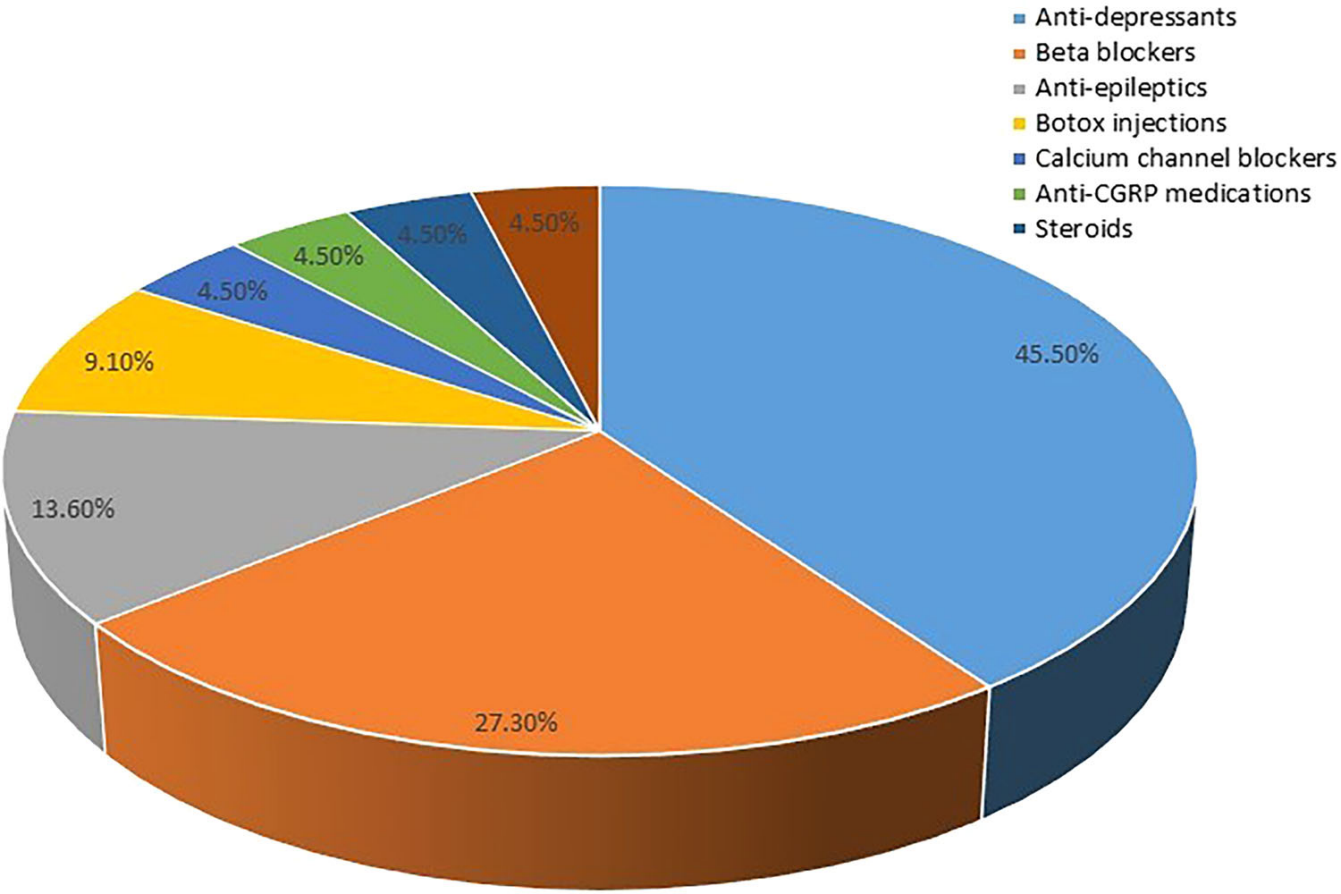
Preferential use of self‐medications for prophylaxis of migraine among participants (*N* = 21).

The medications employed by study participants for the self‐treatment of migraines are presented in Figure 2. These medications were categorized into two main groups: over‐the‐counter (OTC) medications and prescription‐only medications (POMs). Paracetamol and non‐steroidal anti‐inflammatory drugs (NSAIDs) were classified as OTC medications, while paracetamol + codeine, Triptans, Ergot derivatives, and Opioids were categorized as POMs. Analysis of self‐treatment practices revealed a predominant reliance on OTC medications. The majority of study participants utilized paracetamol (70.9%), followed by NSAIDs (43.0%). Among the POMs, paracetamol + codeine was used by 31.5% of participants, Triptans by 6.1%, Ergot derivatives by 1.2%, and Opioids by 1.2%. A small percentage (3.6%) reported using other unspecified medications. These findings indicate that OTC analgesics, particularly Paracetamol and NSAIDs, are the most frequently used agents for migraine self‐treatment within the study population. The preferential use of SM for migraine prophylaxis among the participants (*N* = 21) is shown in Figure 3. Among 21 participants, 45.5% of participants were using antidepressants, followed by 27.3% using beta blockers.

**FIGURE 4:**
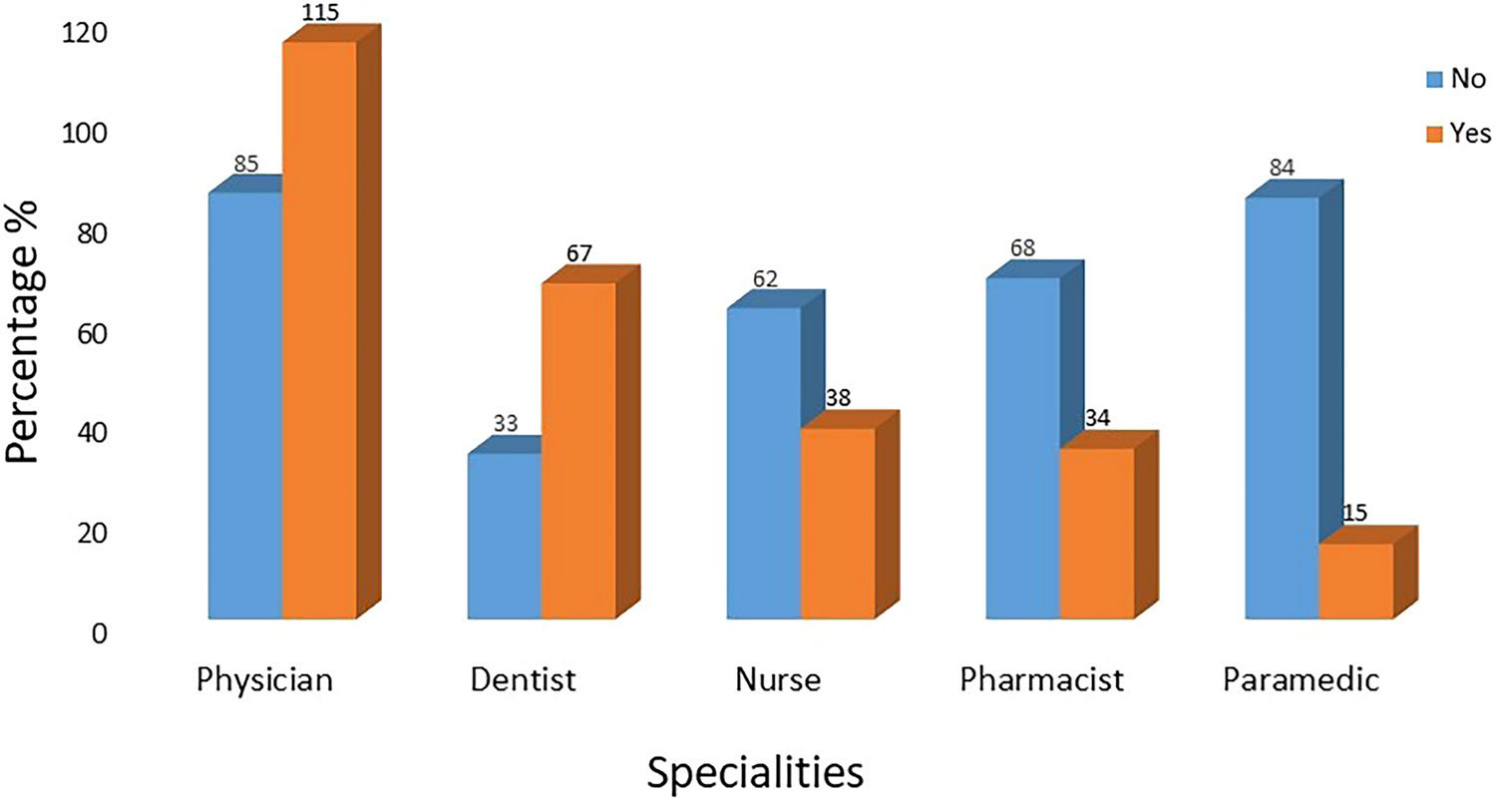
Relative intake of NSAIDs as self‐medication for migraine among different healthcare specialties (*N* = 165).

The study participants used several SM drugs. An interesting correlation was observed between the different specialties that were taking NSAIDs. Statistical analysis showed a statistically significant strong positive correlation between a participant's specialty and NSAIDs intake; χ2(5, *N* = 165) = 25.018, *p* < 0.0005 (Figure 4). There were significant positive correlations between the following SM drugs and reported side effects: paracetamol + codeine use was associated with a high incidence of vomiting and diarrhea. The administration of ergot derivatives demonstrated a significant risk of gastrointestinal side effects, including stomachache and heartburn. The potential side effects of triptans include diarrhea, dizziness, and drowsiness (Table 4).

**TABLE 4:** Significant positive correlations with self‐medications and the reported side effects (analyzed using Chi‐square test).

Drug	Side effect(s)
Paracetamol + codeine	Vomiting; diarrhea
Ergot derivatives	Stomach ache; heartburn
Triptans	Diarrhea; dizziness; drowsiness
Opioids	Vomiting; heartburn
Others	Constipation; dizziness; irritability; syncope

## 4 Discussion

Migraine, a rather complex disease associated with a multitude of syndromes, is considered one of the most prevalent and potentially devastating diseases experienced by HCPs (Albasheer et al. 2016; Straube and Andreou 2019). The results of this study showed that of the 1054 participants, 803 HCPs had headaches, while 219 suffered from a migraine, of which 165 were self‐medicating. Physicians and other HCPs were no exceptions to SM. As migraine headaches are considered one of the most common and potentially devastating diseases experienced by HCPs, SM among those HCPs is highly expected (Kuo et al. 2015).

This study SM rate among migraineur HCPs (75.3%) falls within the high range observed in other studies involving medical students and health professionals globally, aligning closely with findings from Egypt (71%) and Malaysia (69.14% for headache sufferers). This underscores a consistent pattern of high SM practices within the healthcare community worldwide. (Hassan and Koabar 2025; Thiagarajan et al. 2022). A global meta‐analysis reported a pooled SM prevalence of 70.1% among university students. Specifically, they found a much higher prevalence of 97.2% among medical students compared to 44.7% in non‐medical students (Behzadifar et al. 2020). A global study revealed an overall self‐medication prevalence of 49%. Geographically, America showed a higher prevalence at 47.8% (95% CI: 33.6–62) compared to Africa's 41.5% (95% CI: 29.5–53.5). When examining different groups, students exhibited the highest self‐treatment rate at 54.5% (95% CI: 40.8–68.3), while healthcare workers had the lowest, at 35.5% (95% CI: 16–49) (Kazemioula et al. 2022).

HCPs who were more often self‐medicating were more likely to perceive their headache as a migraine and migraine as an illness. Longer durations with more frequent monthly attacks could be attributed to medication‐overuse headaches (Kristoffersen and Lundqvist 2014). In addition, the presence of a family history of migraine was significantly correlated with headache perceived as a migraine, which reflects the genetic component of the disease, supporting the rationale behind considering a positive family history of migraine as a supportive factor in the provisional diagnosis of the disease (Bron et al. 2021; Pelzer et al. 2019).

This study showed that more than half of the HCPs regarded their headache as a disease; however, many did not seek professional attention and instead pursued SM for relief. This study demonstrated that the most common reasons for SM among HCPs were their perception of having sufficient information about the disease and its management, ease of medication access, and a busy schedule. These motivations resonate with patterns observed globally: medical knowledge from self‐experience and studies (55.9%) was a key determinant among Egyptian medical students while Spanish health professionals cited easy access to medications and professional knowledge as significant factors, alongside the mildness of symptoms and previous successful experiences (Cotobal‐Calvo et al. 2025; Hassan and Koabar 2025). Previous studies have shown that the circumstances and environments in which HCPs work predispose them to access medication during their daily routines. Although most HCPs are aware of the risks associated with self‐medication, many seek solace in it, especially when faced with work‐related stress, pressure, discomfort, and anxiety (Fekadu et al. 2020). In accordance with our study, previous studies found that the reasons for SM among HCPs included familiarity with medications, time savings, and drug accessibility (Mohammed et al. 2021; Sado et al. 2017).

Furthermore, Paracetamol and NSAIDs were the most commonly overused self‐medication (SM) drugs for migraines, with NSAIDs being more frequently used by physicians as over‐the‐counter medications in Saudi Arabia. This high reliance on over‐the‐counter analgesics, particularly paracetamol and NSAIDs, is consistent with findings among Egyptian medical students and Spanish health professionals (Cotobal‐Calvo et al. 2025; Hassan and Koabar 2025). In terms of perceived effectiveness, approximately 53.2% of our self‐medicating HCPs reported their medication was effective, which is lower than the high efficacy rates (78% for prescription, 81% for OTC) reported in a Chinese population study(Chang et al. 2021). Similarly, Viticchi et al. reported that 3.57% of physicians who experienced migraines used paracetamol, 10.7% used NSAIDS, 3.57% used unspecified analgesic drugs, and 3.57% used ergotamine derivatives (Viticchi et al. 2018). In this study, these accessible medications use of a prophylactic drug for migraine, such as an antidepressant or beta‐blocker, was not considered by most HCPs. These results were analogous to the findings of Hansen et al., whose study showed that only 9.7% of neurologists and pain specialists currently use migraine prophylaxis, with 52.9% of them using a recommended first‐line prophylaxis (Hansen et al. 2020). Moreover, we found a significant positive correlation between the use of prophylactic agents and the duration of migraine, frequency of attacks, and seeking professional consultation.

Interestingly, our findings illustrated that 78.5% of the HCPs did not encounter any side effects from SM, while only a few experienced adverse effects, nausea and stomachaches being the most common; however, this could be influenced by recall bias. Similarly, a prospective cross‐sectional survey by Sridhar et al. illustrated that many respondents thought SM was safe; however, some reported side effects or complications during SM (Sridhar et al. 2018).

According to the International Classification of Headache Disorders, the term “medication‐overuse headache” (MOH) refers to “headache occurring on 15 or more days per month developing as a consequence of regular overuse of acute or symptomatic headache medication for more than 3 months.” (Kristoffersen and Lundqvist 2014). A significant proportion of self‐medicating HCPs (58.6%) in this study were unfamiliar with this term, with only 21% able to correctly define MOH. This finding suggests a substantial educational gap among HCPs regarding a critical complication of headache management. This low awareness is particularly concerning given the observed high prevalence of SM and the potential for unsupervised medication use to inadvertently lead to MOH. An article by Minen et al. was compatible with our study, showing only 34% awareness among HCPs about the fact that opioids could result in MOH (Minen et al. 2016). This highlights a disparity between HCPs' perception of having sufficient information for self‐management and their actual comprehensive understanding of complex headache conditions and their complications. It may also be recognized as a complication of chronic migraine because it typically occurs in the context of frequent migraine headache attacks (Westergaard et al. 2016). A comprehensive systematic review by Thorlund et al. suggested that compared to other treatments, patients receiving acute migraine treatment, analgesics, and opioids have a higher risk of developing MOH (Thorlund et al. 2016). Another possible risk factor for medication overuse is the progression of episodic to chronic migraine (Bigal et al. 2008). Employed participants with current high‐frequency migraines and medication overuse experienced notable stigma, such as feeling left out or embarrassed due to the limitations from their headaches, which had a negative impact on their self‐confidence and ability to work (Buse et al. 2024). This critical gap in MOH knowledge among HCPs warrants immediate attention through targeted policy interventions and educational initiatives, such as mandatory continuing medical education (CME) modules focused on MOH diagnosis, prevention, and management, to improve both their personal health outcomes and their clinical guidance to patients.

In this study, an interesting correlation was observed between different specialties taking NSAIDs. A previous study reported that NSAIDs pose a risk of upper gastrointestinal bleeding or perforation of varying severity, depending on the specific NSAID and dosage (Doomra and Goyal 2020). NSAIDs can cause serious problems such as heart issues, kidney damage, and stomach ulcers. To reduce this risk, it is important to carefully choose which NSAID to use, identify patients who are more likely to have problems, and monitor them closely while they are taking the medication (Szeto et al. 2020). The literature suggests that nausea, dizziness, vomiting, and constipation are common side effects of codeine plus paracetamol, which corroborates our findings (Kjærsgaard‐Andersen et al. 1990). In addition, triptan toxicity typically manifests as high blood pressure, rapid heart rate, and drowsiness, and ergotamine poisoning often presents as an acute gastrointestinal disturbance accompanied by dizziness and numbness (Robblee et al. 2020).

### 4.1 Limitations and Strength

Owing to the inherent limitations of the surveys, our study has several limitations. The cross‐sectional design provides only a snapshot of the situation at a single point in time. It cannot establish a cause‐and‐effect relationship between SM and its potential consequences, and may not be representative of all HCPs in Riyadh. This study used self‐reported data obtained through an electronic questionnaire. This can be subject to recall or social desirability bias, and inaccuracies in reporting medication use and side effects. A specific limitation stemming from this self‐reported nature is the absence of detailed dosage information for any self‐medicated medications, which restricts a more precise assessment of medication overuse and its clinical implications. This study included a relatively large sample size (1054 HCPs), which increased the statistical power and generalizability of the findings. This study collected data on various aspects of SM, including reasons, medications used, perceived effectiveness, and side effects. This study identified key motivators for SM such as knowledge about the disease, easy access to medications, and busy schedules. This information is crucial for developing targeted interventions.

## 5 Conclusion

In conclusion, migraines can be considered a disabling barrier for healthcare providers, limiting their functionality and quality of life. Several reasons for SM have been shown to be significant, including the perception of having sufficient information and its management, accessible medications, and busy schedules. The most commonly overused SM approaches for migraine are Paracetamol and NSAIDs and classified as over the counter medications in Saudi Arabia. Significant positive correlations were observed between certain SM drugs and the reported side effects. Healthcare professionals' limited understanding of MOH needs immediate attention and implement targeted policies and educational programs, including mandatory continuing medical education (CME) focused on MOH diagnosis, and management. Targeted health education initiatives aimed at raising awareness and promoting self‐knowledge are crucial for mitigating the impact of this issue. Additionally, after the diagnosis of migraine, consultation with a neurologist for personalized management plans, including prophylactic treatment and appropriate analgesics, is strongly recommended.

## Author Contributions

**Bandar Nasser Aljafen**: conceptualization, supervision, writing – review and editing, investigation, validation. **Jodi Mohamad Alkahwaji**: methodology, writing – original draft, data curation. **Sarah Amin Alamoudi**: methodology, writing – original draft, data curation. **Shaimaa Tawfik Jamous**: methodology, writing – original draft, data curation. **Sara Mohammed Almesfer**: writing–original draft, data curation, formal analysis. **Aljohrah Sultan Alanazi**: writing – original draft, data curation, formal analysis. **Fatima Yahya Al‐Aidaros**: writing – original draft, data curation, formal analysis.

## Ethics Statement

The study was conducted in accordance with the Declaration of Helsinki, and approved by the Institutional Review Board of Institutional Review Board (IRB), College of Medicine, King Saud University, Kingdom of Saudi Arabia (IRB Approval No. E‐22‐6683).

## Conflicts of Interest

The authors declare no conflicts of interest

## Peer Review

The peer review history for this article is available at https://publons.com/publon/10.1002/brb3.70724.

## Supporting information




**Supplementary Materials**: brb370724‐sup‐0001‐Supplementarydata1‐1.docx

**Supplementary Materials**: brb370724‐sup‐0002‐Supplementarydata1‐2.docx

**Supplementary Materials**: brb370724‐sup‐0003‐Supplementarydata2‐1.docx

**Supplementary Materials**: brb370724‐sup‐0004‐Supplementarydata2‐2.docx

## Data Availability

All the data generated or analyzed in the current study are included in this article.
