# Supplementary material for: Assessing Self‐medication Practices Among Healthcare Providers With Migraine in Saudi Arabia
Source: Brain Behav. 2025 Aug 22;15(8):e70724. doi: 10.1002/brb3.70724 (PMC12373708; doi:10.1002/brb3.70724)
Supplement: Supplementary file 1 — Supplementary Materials: brb370724‐sup‐0001‐Supplementarydata1‐1.docx [file BRB3-15-e70724-s004.docx]

Assessing Self-medication Practices among Healthcare Providers with Migraine in Saudi Arabia

Bandar Nasser Aljafen^1*^, Jodi Mohamad Alkahwaji^2^, Sarah Amin Alamoudi^2^, Shaimaa Tawfik Jamous^2^, Sara Mohammed Almesfer^2^, Aljohrah Sultan Alanazi^2^ and Fatima Yahya Al-Aidaros^2^

^1 Neurology Unit, Department of Medicine, College of Medicine, King Saud University, Riyadh, 11461, Saudi Arabia; baljafen@ksu.edu.sa (BNA)^

^2 College of Medicine, Dar Aluloom University, Riyadh, Saudi Arabia; jodialkah@gmail.com (JMA) alamoudisarah9@gmail.com (SAA), Shaimaa.ja25@gmail.com (STJ), sarahalmesfer@gmail.com (SMA), aljohrah665@gmail.com (ASA), Fatima.y1998@gmail.com (FYA).^

^* Correspondence: baljafen@ksu.edu.sa^

**Table S1**: Demographic characteristics and diagnosis of migraine (*N* = 1054)

| Characteristic | All Participants (*N* = 1054) |
| --- | --- |
| Age, years(Mean ± SD) | 34.04 ± 8.2 |
| Sex N(%) |  |
| Male | 490 (46.5%) |
| Female | 564 (53.5%) |
| Specialty N(%) |  |
| Physician | 339 (32.2%) |
| Dentist | 67 (6.4%) |
| Nurse | 343 (32.5%) |
| Pharmacist | 59 (5.6%) |
| Paramedic | 246 (23.3%) |
| Type of Institute N(%) |  |
| Primary Healthcare Center | 288 (27.3%) |
| Secondary Healthcare Center | 249 (23.6%) |
| Tertiary Healthcare Center | 517 (49.1%) |
| Had headache in the past 3 months? N(%) |  |
| **Yes** | **803 (76.2%)** |
| No | 251 (23.8%) |
| Experienced the following symptoms (nausea, photophobia, disabling headache) N(%) |  |
| **≥ 2 Symptoms** | **219 (20.8%)** |
| 1 Symptom | 313 (25.7%) |
| None of these symptoms | 271 (29.7%) |
| N/A (Never had headache) | 251 (23.8) |

**Table S2**: Correlation between different factors and migraine

|  | Regard headache as migraine? | | Sought medical professional treatment? | | | Taking self-medication for migraine | | Regard migraine as illness | | Duration of experiencing migraine (years) | | Frequency of attacks/ 3 months | |
| --- | --- | --- | --- | --- | --- | --- | --- | --- | --- | --- | --- | --- | --- |
|  | *r_s_* | *P** | *r_s_* | *P** | | *r_s_* | *P** | *r_s_* | *P** | *r_s_* | *P** | *r_s_* | *P** |
| Age (years) | 0.163 | 0.037 | 0.158 | 0.042 | | NS | | NS | | NS | | NS | |
| Specialty | -0.204 | <0.001 | NS | | | -0.262 | <0.001 | NS | | NS | | NS | |
| Duration of Experiencing Migraine (years) | 0.402 | <0.001 | 0.267 | 0.001 | | NS | | 0.254 | 0.001 | NA | | NS | |
| Frequency of Attacks/3 months | 0.266 | 0.001 | 0.214 | 0.006 | | NS | | 0.239 | 0.002 | NS | | NA | |
| Family History of Migraine | 0.186 | 0.014 | NS | | | NS | | NS | | NS | | NS | |
| Frequency of Self-Medication/month | 0.169 | 0.033 | NS | | | NS | | 0.185 | 0.019 | 0.193 | 0.015 | 0.500 | <0.001 |
| Appreciating Effectiveness of Self-Medication | -0.274 | 0.001 | NS | | | NS | | -0.300 | <0.001 | NS | | -0.222 | 0.005 |
| Self-Medication for Prophylaxis | NS | | 0.405 | | <0.001 | NS | | NS | | 0.200 | 0.013 | 0.162 | 0.043 |

* *r_s_*, Correlation Coefficient (Spearman rho); *p* <0.05 considered as significant.

NS: non-significant; NA: not applicable
