# Supplementary material for: Assessing Self‐medication Practices Among Healthcare Providers With Migraine in Saudi Arabia
Source: Brain Behav. 2025 Aug 22;15(8):e70724. doi: 10.1002/brb3.70724 (PMC12373708; doi:10.1002/brb3.70724)

Assessing Self-medication Practices among Healthcare Providers with Migraine in Saudi Arabia

Bandar Nasser Aljafen^1*^, Jodi Mohamad Alkahwaji^2^, Sarah Amin Alamoudi^2^, Shaimaa Tawfik Jamous^2^, Sara Mohammed Almesfer^2^, Aljohrah Sultan Alanazi^2^ and Fatima Yahya Al-Aidaros^2^

^1 Neurology Unit, Department of Medicine, College of Medicine, King Saud University, Riyadh, 11461, Saudi Arabia; baljafen@ksu.edu.sa (BNA)^

^2 College of Medicine, Dar Aluloom University, Riyadh, Saudi Arabia; jodialkah@gmail.com (JMA) alamoudisarah9@gmail.com (SAA), Shaimaa.ja25@gmail.com (STJ), sarahalmesfer@gmail.com (SMA), aljohrah665@gmail.com (ASA), Fatima.y1998@gmail.com (FYA).^

^* Correspondence: baljafen@ksu.edu.sa^

**Figure S1**: Diagram illustrating selection of study participants

Survey distributed to 1196 health care providers

25 participants were excluded who did not consent to fulfill the survey

1171

117 were excluded who were not actively engaged in the survey


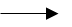


1054

251 participants were excluded who did not complain of headaches for past 3 months


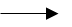


803


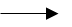


584 participants were excluded who did not fulfill the ID-Migraine Screening test

219


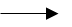


54 participants were excluded who did not report self-medicating for their migraine headaches

165

**Figure S2**: Response of participants to survey question about definition of “medication-overuse headache” (*N* = 21).


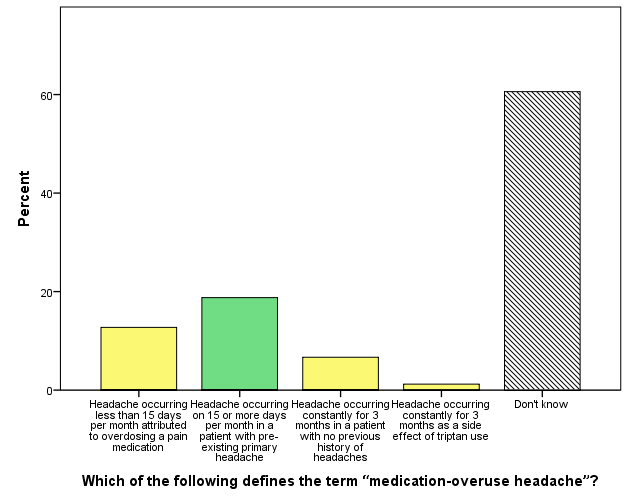

Supplement: Supplementary file 3 — Supplementary Materials: brb370724‐sup‐0003‐Supplementarydata2‐1.docx [file BRB3-15-e70724-s001.docx]
